# Management of frontal sinus trauma: a retrospective study of surgical interventions and complications

**DOI:** 10.1186/s40902-024-00414-z

**Published:** 2024-01-18

**Authors:** InKyeong Kim, Jeong-Mo Kim, Jiha Kim, Seung Jin Lee, Eui-Cheol Nam

**Affiliations:** 1 Department of Neurosurgery, Kangwon National University Hospital, Chuncheon, Korea; 2 Department of Oral & Maxillofacial Surgery, Kangwon National University Hospital, Baengnyeong-ro 156, Chuncheon, Gangwon-do 24289 Korea; 3 Department of Neurosurgery, School of Medicine, Kangwon National University, Chuncheon, Korea; 4 Department of Otolaryngology, School of Medicine, Kangwon National University, Chuncheon, Korea

**Keywords:** Frontal sinus fracture, Obliteration, Cranialization, Complications, Infection

## Abstract

**Background:**

Frontal sinus injuries are relatively rare among facial bone traumas. Without proper treatment, they can lead to fatal intracranial complications, including meningitis or brain abscesses, as well as aesthetic and functional sequelae. The management of frontal sinus injuries remains controversial, with various treatment methods and outcomes being reported. This article describes the clinical characteristics, surgical methods, and outcomes among 17 patients who underwent surgery for frontal sinus injury and related complications.

**Case presentation:**

We retrospectively included 17 patients who underwent surgery for frontal sinus injury and its related complications at the Kangwon National University Hospital between July 2010 and September 2021. Among them, six underwent simple open reduction and fixation of the anterior wall, eight underwent sinus obliteration, and three underwent cranialization. Two patients who underwent sinus obliteration died due to infection-related complications. The patient who underwent cranialization reported experiencing chronic headache and expressed dissatisfaction regarding the esthetic outcomes of the forehead. Except for these three patients, the other patients achieved satisfactory esthetic and functional recovery.

**Conclusion:**

Active surgical management of frontal sinus injuries is often required owing to the various complications caused by these injuries; however, several factors, including the fracture type, clinical presentation, related craniomaxillofacial injury, and medical history, should be considered while formulating the treatment plan. Surgical treatment through the opening of the frontal sinus should be actively considered in patients with severely damaged posterior wall fractures and those at risk of developing infection.

## Background

The incidence of frontal sinus fracture is relatively rare, accounting for only 2–15% of cases of maxillofacial trauma, given the dome-shaped anterior wall of the sinus and the presence of thick cortical bone [1–5]. Frontal sinus injuries are usually caused by high-velocity blunt force trauma; therefore, they are frequently associated with adjacent maxillofacial and intracranial injuries as well as neurosurgical complications [4–9]. Treatment strategies that do not adequately address frontal sinus fractures can lead to serious infections and long-term structural complications; therefore, accurate examination and active treatment are mandatory [10, 11]. Meningitis or cerebritis, cerebrospinal fluid (CSF) rhinorrhea, and pneumocephalus are complications that occur during the acute period, whereas chronic frontal sinusitis, mucocele and mucopyocele, meningitis, brain abscess, and protrusion or depression of the forehead are delayed complications [12, 13]. Accordingly, frontal sinus injuries are a common concern for neurosurgeons, oral and maxillofacial surgeons, and otolaryngologists [12]. The type of fracture, degree of posterior wall injury, nasofrontal outflow tract (NFOT) injury, severity of the accompanying cerebral trauma, neurological status, and CSF leakage are important factors that are considered when selecting the surgery modality [3, 9, 12, 14, 15]. Cranialization, obliteration, and open reduction and internal fixation can be performed depending on the site and extent of the injury. Minimally invasive surgeries using transnasal or transorbital endoscopic techniques have been increasingly used in recent years [7, 16, 17]. This study aimed to describe the diagnoses, surgical methods used according to the fracture pattern, outcomes, and complications of 17 patients who underwent surgery for frontal sinus fracture over the past 11 years.

## Case presentation

### Patients and methods

This retrospective study was reviewed and approved by the Institutional Review Board of the Kangwon National University Hospital (KNUH 2022-02-030). Thirty-eight patients presented to the Kangwon National University Hospital with frontal sinus injury and its related complications between July 2010 and September 2021. Among them, 21 patients with linear fractures or minimal displacement of the frontal sinus wall were treated conservatively (Table 1). Accordingly, we included 17 patients who underwent surgical treatment. Among them, 16 patients had severely displaced or comminuted fractures of the frontal sinus wall with contour deformity or CSF leakage. One patient presented sinogenic meningitis as a complication of a previous frontal sinus injury. We retrospectively analyzed the causes, classification of fractures, clinical findings, the presence of other accompanying facial fractures, surgical methods, and complications. Diagnosis and treatment were performed in close cooperation with each clinical department. The patients initially underwent neurological evaluation, followed by appropriate treatments, including craniotomy, if necessary. The presence of CSF rhinorrhea was confirmed by otolaryngologists, who evaluated the pre- and postoperative status of the damaged frontal sinus. In case endoscopic examination of the nasal cavity revealed persistent watery rhinorrhea and changes in the outflow of watery rhinorrhea were observed by pressing the jugular vein, the patient was clinically judged to have CSF rhinorrhea. Oral and maxillofacial surgery was performed via the opening of the damaged frontal sinus. The surgery was performed in collaboration with the neurosurgery department if the patient had persistent or suspected CSF leakage.

**Table 1:** Pattern and demographics of patients who received conservative management

			Value
Total no. of patients			21
Average age, median year (range)			39 (15–77)
Follow-up duration, median month (range)			61.4 (1–145)
Sex, no	Male		20
	Female		1
Type of fracture	Anterior wall	linear	2
		Minimal	5
	Posterior wall (linear)		2
	Combined anterior and posterior wall	Linear	8
		Minimal	4
Cause of injury	Road traffic accident		1
	Bicycle traffic accident		5
	Interpersonal assault		4
	Slip		5
	Fall		3
	Sports injury		2
	In car traffic accident		1
Neurosurgical problem	Traumatic EDH		2
	Traumatic SDH		1
	Falx SDH		1
Associated fracture	Orbital (roof, medial wall, floor) fracture		14
	Nasal bone fracture		4
	ZMC fracture		6
	Maxillary anterior wall fracture		3
	NOE fracture		1
	LeFort I, II fracture		1

*ZMC* zygomaticomaxillary complex, *NOE* naso-orbito-ethmoid, *EDH* epidural hematoma, *SDH* subdural hematoma

The fractures were classified based on the axial view of facial computed tomography (CT) images; moreover, the surgical approach was determined based on the clinical and radiological findings. The incision was extended along the laceration in two patients with forehead lacerations and fractures confined to the anterior wall. The bicoronal surgery approach was used in the remaining 15 patients. Surgeries are performed via three approaches at our hospital: open reduction and fixation, sinus obliteration, and cranialization. Six patients with fractures confined to the anterior wall of the frontal sinus underwent simple open reduction and fixation. Here, the depressed anterior wall was restored using a bone hook, and the defective wall was repaired using absorbable mesh or porous polyethylene materials. Subsequently, fixation was performed using a titanium metal plate and screw. Seven patients with combined fractures of the anterior and posterior walls and one patient with sinogenic meningitis underwent sinus obliteration. Here, the damaged posterior wall was exposed by elevating the damaged or osteotomized anterior wall. Next, the fractured posterior wall was reduced, and the mucous membrane of the invaded sinus was removed. Obliteration was performed using a collagen sponge, hydroxyapatite cement, or autologous cancellous bone. The anterior wall was restored and fixed using a titanium metal plate, absorbable mesh, or porous polyethylene. Three patients with significantly displaced fractures of the posterior wall and persistent CSF leakage underwent cranialization. Here, frontal craniotomy and dural repair were initially performed by a neurosurgeon, followed by removal of the posterior wall of the frontal sinus and the remaining mucous membrane by an oral and maxillofacial surgeon. Hydroxyapatite cement or a collagen sponge was used to close the NFOT. Table 2 summarizes the type of fracture, cause of injury, associated fractures, clinical presentation, surgical method, and complications associated with frontal sinus injuries in each patient.

**Table 2:** Summary of patient details

Patient (sex/age)	Cause of injury	Associated fracture	Neurosurgical problem	Type of frontal sinus fracture		Surgical method and material	Neurosurgical intervention	Complication & current status
				Isolated anterior wall	Combined anterior and posterior wall			
1 (F/50)	Road traffic accident	Both NOE, right ZMC		Comminuted		ORIF with metal plate		None
2 (M/15)	Fall			Comminuted		ORIF with absorbable mesh		None
3 (M/14)	Sports injury			Displaced		ORIF with absorbable mesh		None
4 (M/45)	Chainsaw injury			Comminuted		ORIF with PPM		None
5 (M/23)	Interpersonal assault	Nasal bone		Displaced		ORIF with absorbable mesh		None
6 (M/59)	Chainsaw injury	Orbital floor		Comminuted		ORIF with metal plate and PPM		None
7 (M/38)	Fall	Left orbital roof, medial wall, floor and maxilla			Anterior wall comminuted, posterior wall linear	Sinus obliteration with collagen sponge		None
8 (M/23)	Sports injury	Both orbital roof and medial wall			Anterior wall comminuted, sinus floor comminuted, posterior wall linear	Sinus obliteration with collagen sponge		None
9 (M/46)	Fall	Both maxilla, right orbital roof	CSF rhinorrhea, traumatic SDH		Anterior wall comminuted, sinus floor comminuted, posterior wall comminuted	Sinus obliteration with HAC	Exploration of dural injuries	None
10 (M/51)	Fall	LeFort I and II and right ZMC, nasal bone			Anterior wall comminuted, sinus floor comminuted, posterior wall comminuted	Sinus obliteration with HAC		None
11 (M/60)	Fall	Right orbital roof, left maxilla, nasal bone	CSF rhinorrhea, traumatic ICH		Anterior wall comminuted, sinus floor comminuted, posterior wall comminuted	Cranialization	Exploration of dural injuries	None
12 (M/43)	Interpersonal assault (hammer injury)	Right orbital roof	CSF rhinorrhea		Anterior wall comminuted, sinus floor comminuted, posterior wall comminuted	Cranialization	Craniotomy and dural repair	None
13 (M/34)	Fall	Right temporal bone, ZMC and NOE, left orbital medial wall, nasal bone	CSF rhinorrhea, traumatic EDH, SDH		Anterior wall comminuted, sinus floor comminuted, posterior wall comminuted	Sinus obliteration with HAC	Craniotomy and dural repair	None
14 (M/49)	Road traffic accident	Both temporal bone, left maxilla, nasal bone	CSF rhinorrhea, traumatic SAH, SDH, EDH		Anterior wall comminuted, sinus floor comminuted, posterior wall comminuted	Cranialization	Craniotomy and EDH removal, dural repair	Intermittent headache, forehead contour irregularity
15 (M/67)	Slip	Left orbital roof	Traumatic EDH		Anterior wall comminuted, sinus floor comminuted, posterior wall comminuted	Sinus obliteration with HAC		None
16 (M/88)	Pedestrian accident	Both NOE, right maxilla, left ZMC	Traumatic SAH, SDH		Anterior wall comminuted, sinus floor comminuted, posterior wall comminuted	Sinus obliteration with HAC	Exploration of dural injuries	Brain abscess, death
17 (M/44)	History of previous frontal sinus fracture		CSF rhinorrhea		Anterior wall, sinus floor, posterior wall defect	Sinus obliteration with autologous cancellous bone	Craniotomy and dural repair	Recurrent CSF leakage and meningitis, death

*NOE* naso-orbito-ethmoid, *ZMC* zygomaticomaxillary complex, *ORIF* open reduction and internal fixation, *CSF* cerebrospinal fluid, *SDH* subdural hematoma, *ICH* intracerebral hemorrhage, *EDH* epidural hematoma, *SAH* subarachnoid hemorrhage, *PPM* porous polyethylene material, *HAC* hydroxyapatite cement

### Results

**Fig. 1:**
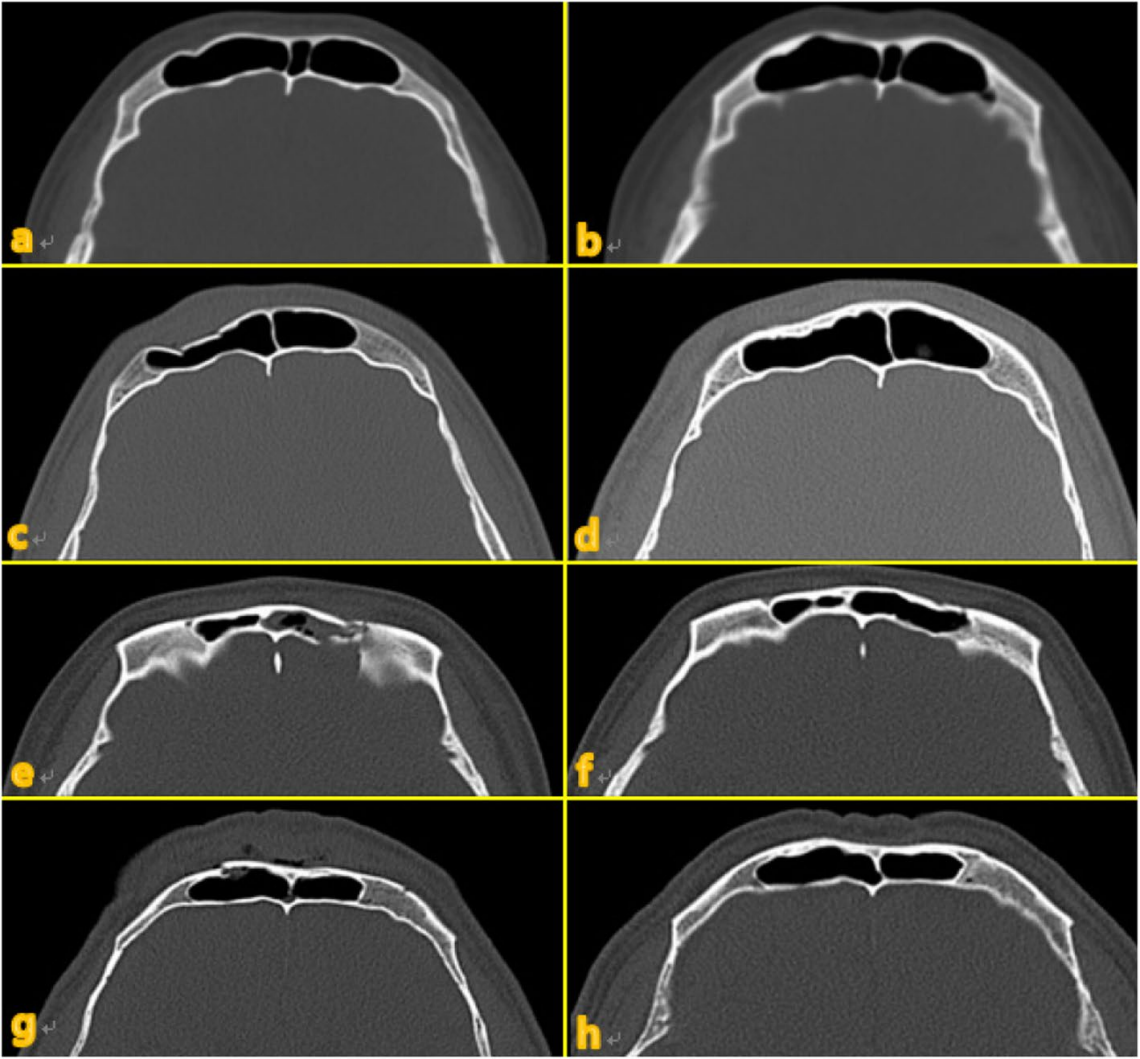
**a**, **c**, **e**, and **g** Computed tomographic imaging at the time of injury. **b**, **d**, **f**, and **g** Post-injury images at 9 years and 9 months, 6 years and 7 months, 1 year and 8 months, and 4 years and 11 months, respectively

All 21 patients who received conservative management achieved good treatment outcomes without any adverse complications. Specifically, some patients showed spontaneous improvement in the outline of the displaced fracture area over time on CT imaging (Fig. 1).

All but one of the 17 patients who underwent surgery were male. The etiology of frontal sinus fracture was falls in seven patients, road traffic accidents in three patients, sports injuries in two patients, chainsaw injuries in two patients, and interpersonal assaults in two patients. One patient presented sinogenic meningitis as a complication of surgery for frontal sinus fracture performed 20 years prior. The ages of the 17 patients ranged from 15 to 88 years (mean, 44.1 years); moreover, the mean follow-up duration was 39.6 months (range, 2–109 months). Forehead lacerations, CSF rhinorrhea, and intracranial hemorrhage due to head trauma were observed in five, six, and six patients, respectively. The patient with sinogenic meningitis showed altered consciousness and CSF rhinorrhea as well as formation of a brain abscess, extensive pneumocephalus, and defects in the floor and posterior wall of the frontal sinus. Two patients developed diplopia, whereas one patient reported loss of vision. One or more adjacent maxillofacial fractures were observed in 13 patients, with most of these fractures being surgically corrected simultaneously. Isolated fractures of the anterior wall of the frontal sinus were observed in six patients; additionally, none of the patients had isolated fractures of the posterior wall. Combined fractures of the anterior and posterior walls were observed in 10 patients. Among them, nine patients showed sinus floor fracture, while one patient had a through-and-through fracture, which is the most severe type of penetrating fracture.

Among the 16 patients with trauma, all 6 patients with frontal sinus fractures involving only the anterior wall showed satisfactory postoperative recovery, with no clinical or radiological complications. Among the 10 patients with combined anterior and posterior wall fracture, forehead deformity and chronic headache as sequelae of cranialization were observed in one patient; further, one patient died due to complications of intracranial infection following sinus obliteration. The remaining eight patients showed satisfactory recovery without any unusual sequelae or complications. The patient with sinogenic meningitis underwent sinus obliteration; however, craniotomy and dural repair were additionally performed due to the recurrence of intracranial infection. However, the patient’s condition did not improve, and he eventually died.

**Fig. 2:**
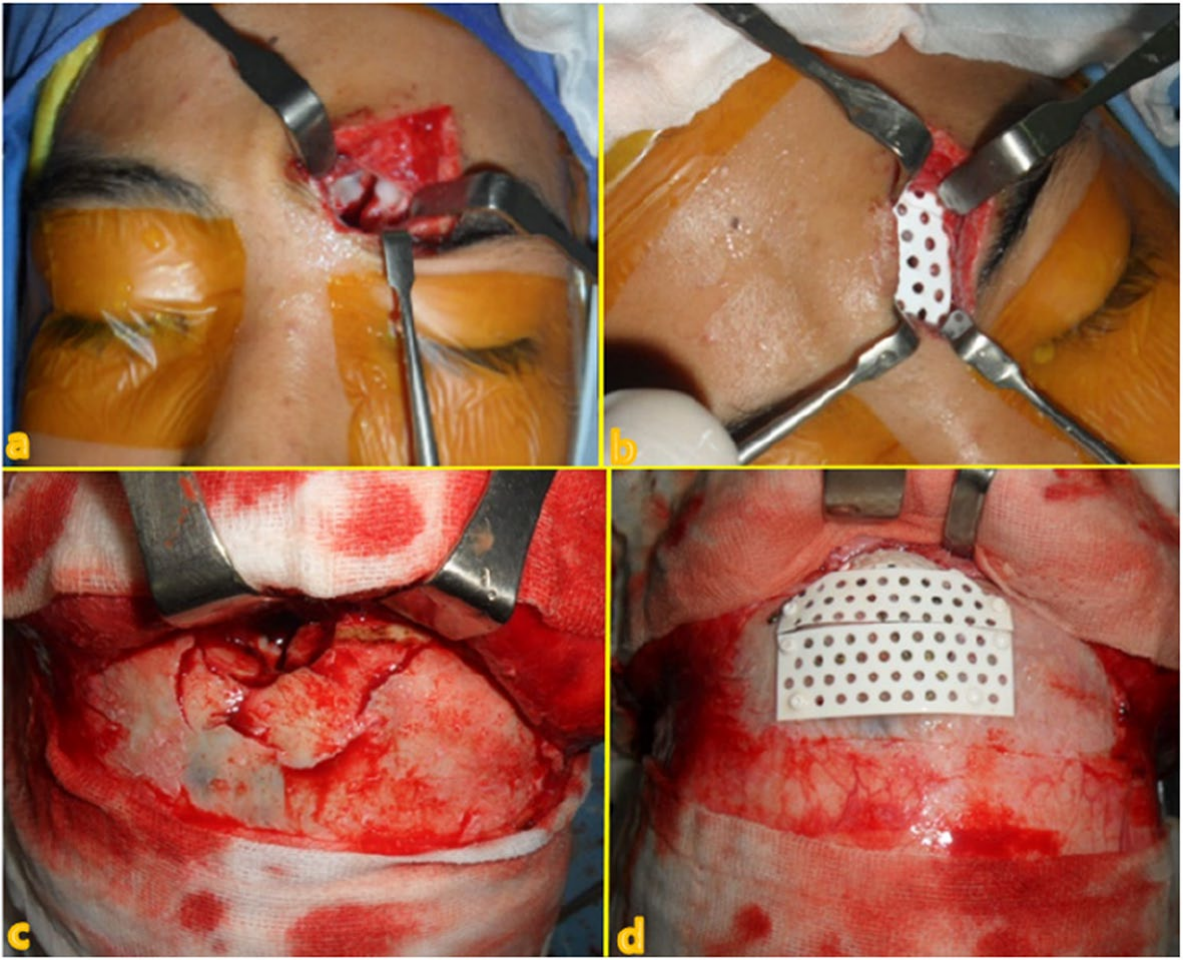
An absorbable mesh was used as the repair material. **a** and **b** Approach via the forehead laceration. **c** and **d** Bicoronal approach

**Fig. 3:**
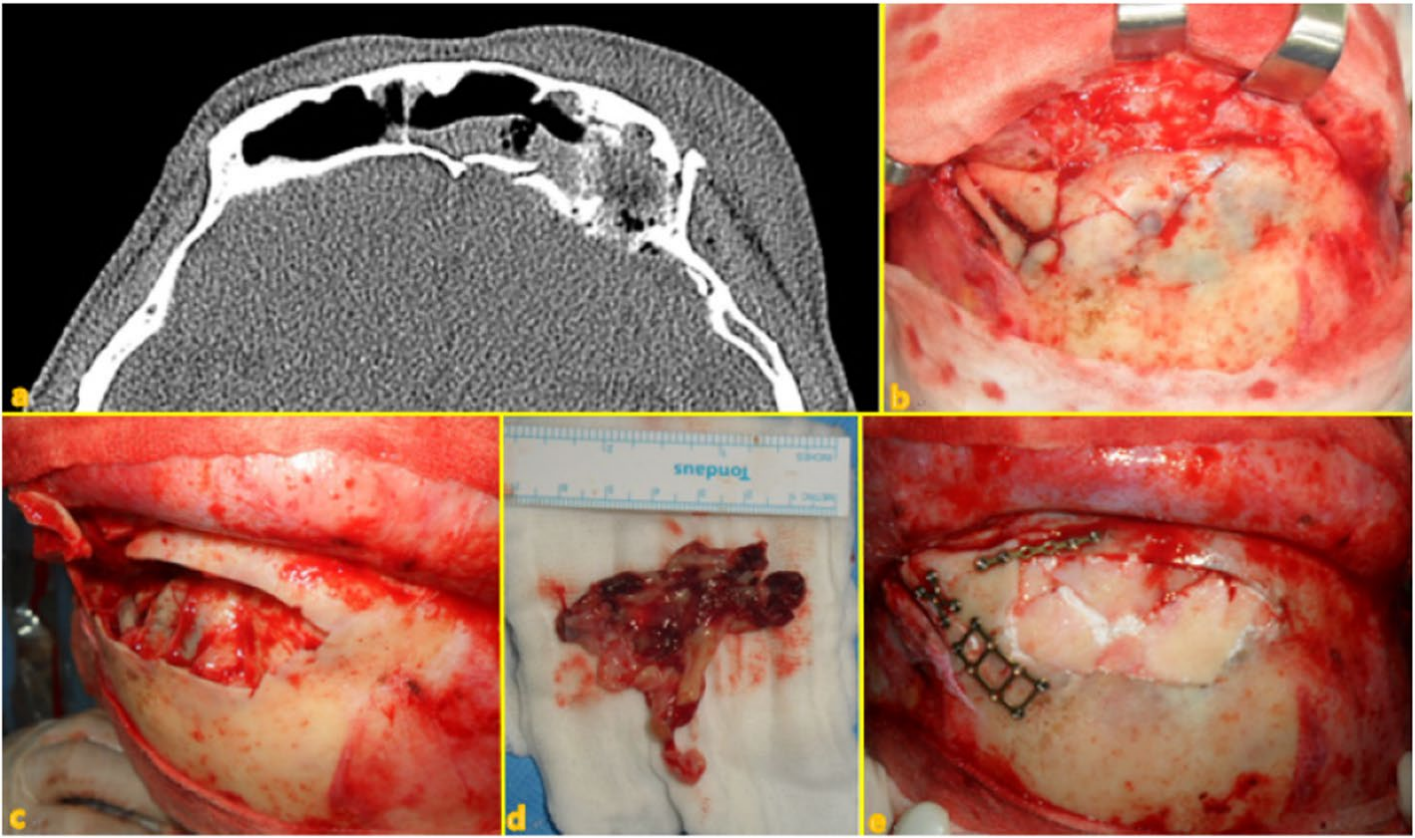
**a** and **b** Combined anterior and posterior wall fracture of the frontal sinus. **c** and **d** Osteotomized and elevated anterior wall of the frontal sinus and exposed posterior wall. The mucous membrane of the invaded sinus was removed. **e** Sinus obliteration performed using hydroxyapatite cement and anterior wall repositioning

**Fig. 4:**
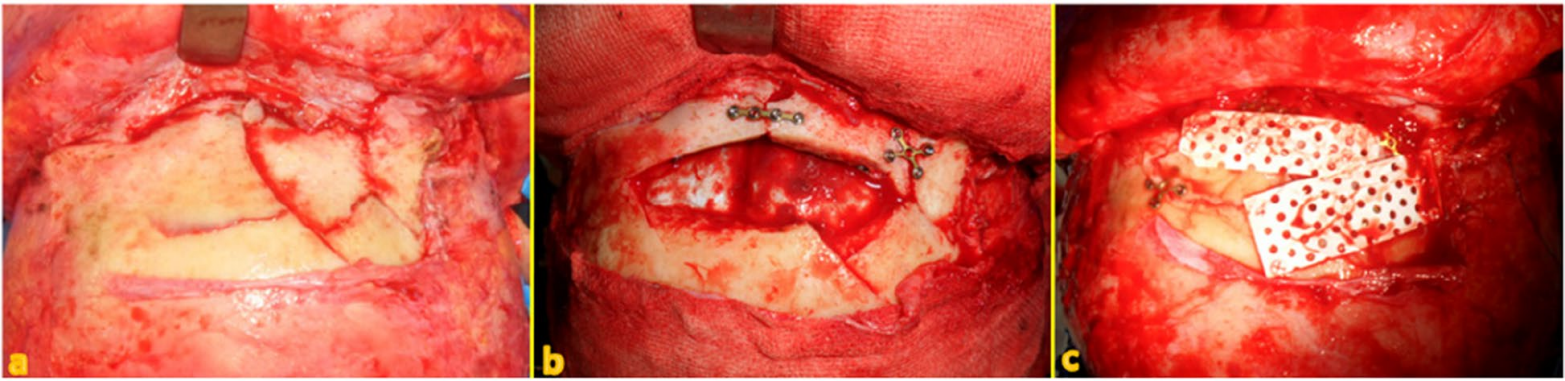
**a** and **b** Osteotomized and elevated anterior wall of the frontal sinus. Removed posterior wall and exposed dura. **c** Repositioned anterior wall

**Fig. 5:**
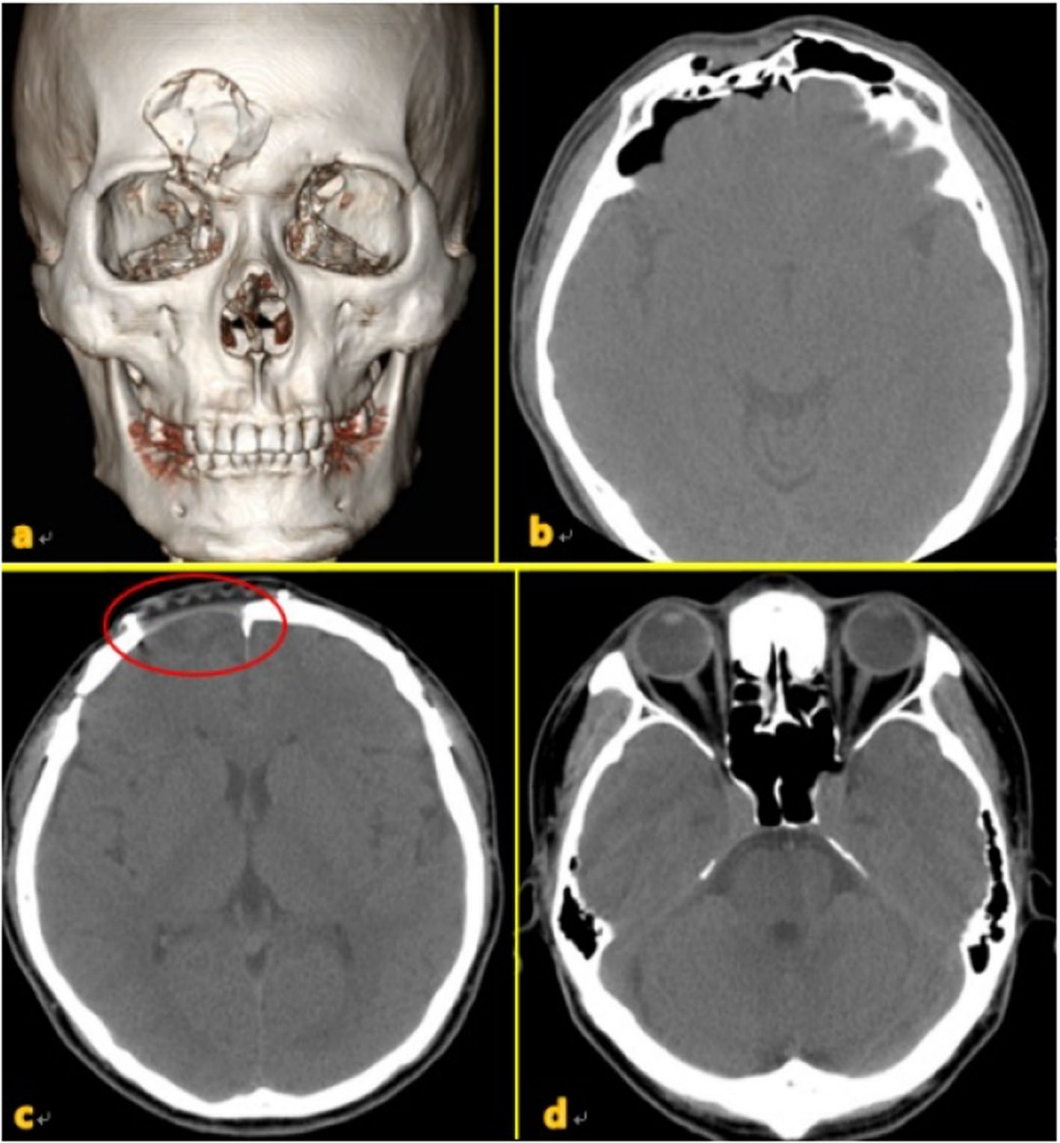
**a** and **b** Through-and-through fracture caused by a hammer impact. Pneumocephalus can be observed on the image. **c** and **d** CT image acquired at 1 year postoperatively shows anterior expansion of the frontal lobe (red circle). CT, computed tomography

**Fig. 6:**
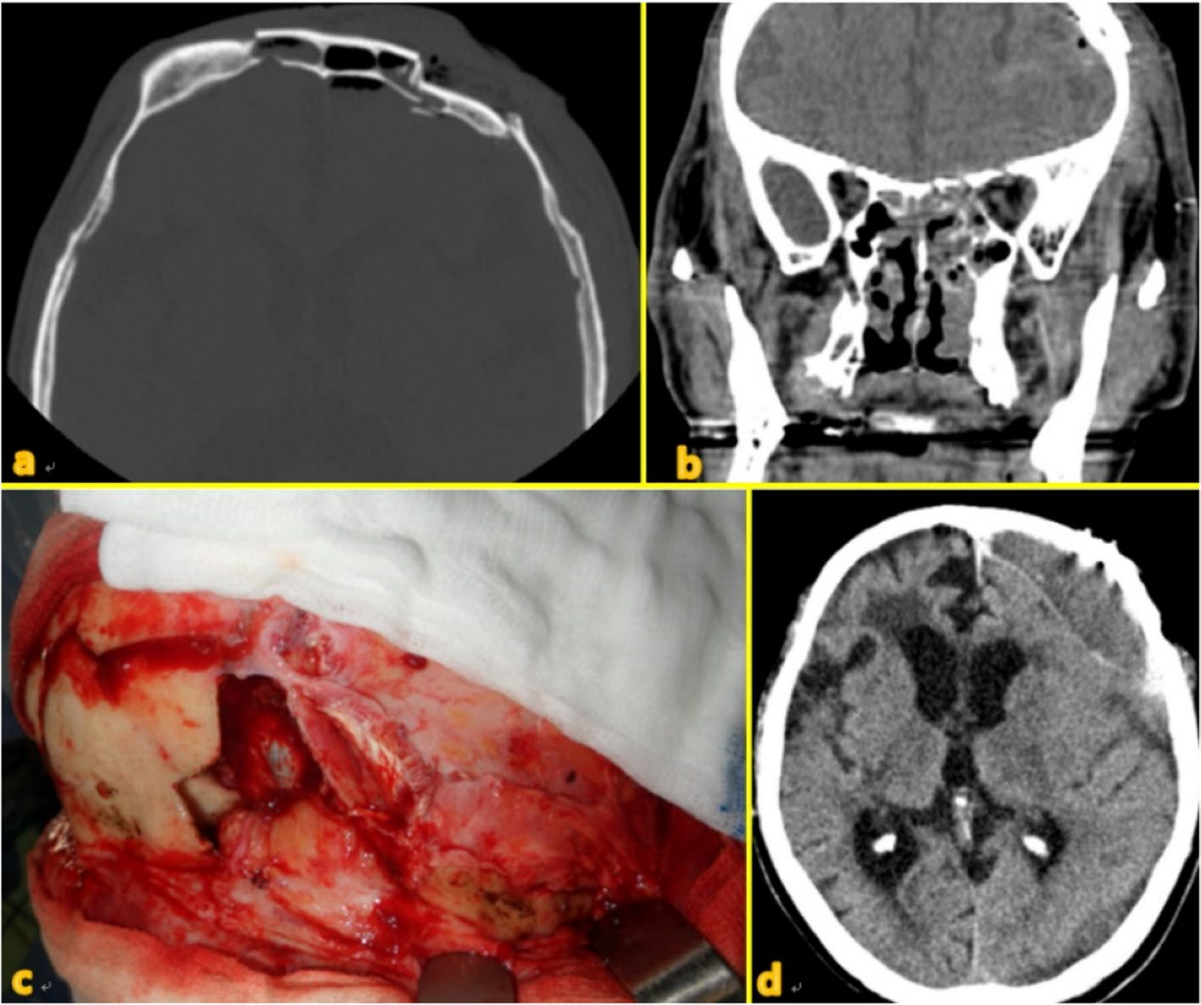
**a** and **b** Combined fracture of the anterior and posterior wall of the frontal sinus. Pneumocephalus can be observed on the image. **c** The supraorbital bone fragment was elevated, and the dura was exposed. **d** A subdural abscess had developed in the left hemisphere at 1 postoperative year

**Fig. 7:**
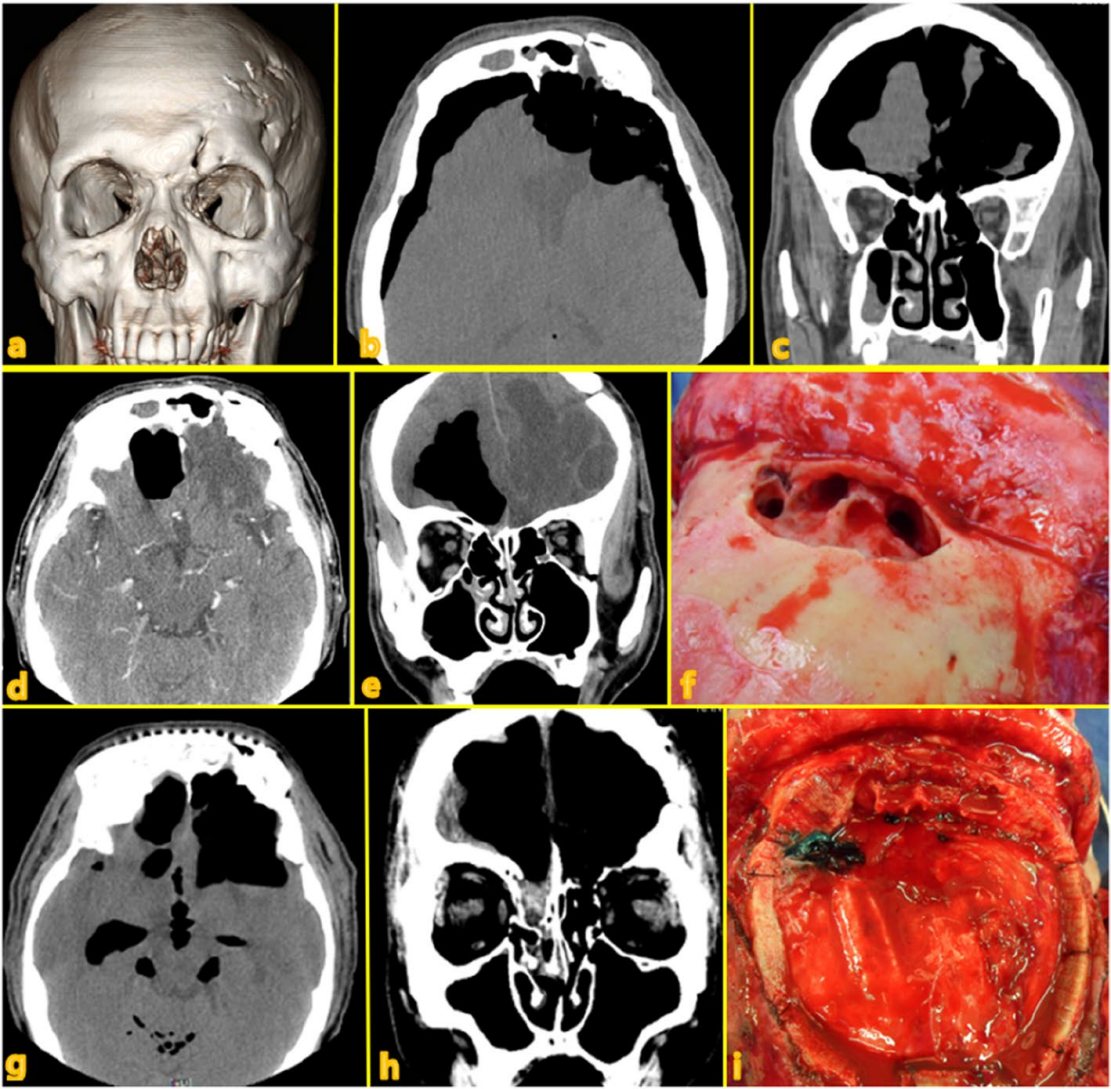
**a**–**c** CT image acquired at the first visit. Medical history of craniotomy, extensive pneumocephalus, defects in the posterior wall and floor of the frontal sinus, and channel formation between the brain and paranasal sinus. The patient refused surgery at this time and was discharged. **d** and **e** CT scan acquired during the second visit. Intracranial abscess formation and pneumocephalus can be observed. **f** The osteotomized anterior wall of the frontal sinus is elevated, and the eroded posterior wall can be observed. Meticulous debridement of the mucosal membrane was performed. The formed channel was identified through the eroded posterior wall. **g** and **h** CT scan image acquired during the third visit (four postoperative weeks) shows increased pneumocephalus. **i** Craniotomy and dural repair were performed. CT, computed tomography

Seven different cases of frontal sinus fracture were included: repair of the anterior frontal sinus with an absorbable mesh via an existing laceration and bicoronal approach (Fig. 2), anterior frontal sinus repair using a metal plate and sinus obliteration through application of hydroxy apatite cement (Fig. 3), cranialization and repositioning of the anterior frontal sinus using a metal plate and an absorbable mesh (Fig. 4), cranialization and reconstruction of the anterior frontal sinus using porous polyethylene material (Fig. 5), brain abscess after sinus obliteration (Fig. 6), and sinus obliteration using auto-iliac cancellous bone in a patient with sinogenic meningitis (Fig. 7).

### Discussion

The therapeutic goals of frontal sinus surgery are protection of the intracranial contents, correction of CSF leakage, prevention of infections and complications, and restoration of functional and esthetic features [18, 19].

Linear or minimally displaced isolated fractures of the anterior wall can be conservatively managed via periodic observation [3, 5, 20, 21]. Moderate-to-severe displacement of isolated fractures of the anterior wall leads to facial deformities; therefore, such cases require open reduction and internal fixation [22, 23]. Isolated fractures of the posterior wall are relatively rare; contrastingly, combined anterior–posterior wall fractures are the most common type of frontal sinus fractures [6, 8, 9, 15, 23]. The posterior wall is relatively thinner than the anterior wall. In case of damage to the posterior wall, CSF may leak via the damaged dura mater if there is an intracranial injury given its direct anatomic relationship with the intracranial space and skull base [7]. Formation of cysts occurs when fractures cause mucosal malposition or entrapment. Subsequently, these cysts secrete mucus and exert pressure on the wall. Erosion of the surrounding bone and mucocele or mucopyocele formation may occur, which may cause intracranial infection, including brain abscess or meningitis, in severe cases [10, 24, 25]. Fracture of the posterior wall can cause narrowing or obstruction of the NFOT, which can lead to disruption of the mucociliary clearance system, with impairment of aeration and drainage of the frontal sinus [8, 23]. This can lead to inflammation of the lining of the mucous membrane, which increases the risk of frontal sinusitis or mucocele formation [6–8, 24, 26, 27]. Therefore, the degree of posterior wall displacement is an important criterion for selecting a treatment method. In our study, the degree of posterior wall damage and CSF leakage were used as the main indicators for surgery.

Conservative management with periodic observation is recommended for linear or minimally displaced fractures of the posterior wall in the absence of dural tears or CSF leakage [2, 5, 7, 26]. In case CSF leakage is observed, a conservative approach is initially pursued with bed rest. Antibiotics and decongestants are administered for 2 to 7 days; further, lumbar drainage may be performed if necessary. No further treatment is required if the CSF leakage resolves spontaneously. However, if it persists, sinus obliteration or cranialization should be considered [7, 23, 28].

Significantly displaced, comminuted fractures of the posterior wall or prolonged CSF leakage is considered indications for cranialization [2, 5, 9, 29]. Cranialization involves circumvention of the sinus cavity by removing the posterior wall of the frontal sinus through a bifrontal craniotomy. Therefore, the brain is allowed to expand into the cranialized sinus, and the sinus space is incorporated into the intracranial space. It effectively reduces intracranial morbidity by preventing CSF leakage or infection due to posterior frontal sinus fractures [6, 7, 23, 26].

In the absence or resolution of CSF leakage, sinus obliteration can be considered as an alternative treatment for severely displaced posterior wall fractures, which are conventionally treated through cranialization. Sinus obliteration is a surgical procedure wherein the sinus cavity is completely filled with avascular material to prevent mucus or CSF leakage into the nasal cavity due to retrograde infection [3, 26]. Various materials have been utilized for sinus obliteration, including autologous cancellous bone, autologous fat, pericranial flap, allogenic or heterogenic bone, and synthetic materials. Doonquah et al. considered autogenous tissue as the gold standard for sinus obliteration [6]. Although autologous cancellous bone is readily available and allows reliable CT monitoring, the required amount may not be used since some degree of donor site morbidity occurs when the tissue is harvested [4, 17]. Moreover, autologous fat has the advantage of being resistant to infection, with slow absorption and gradual replacement by fibrous tissue [10, 30]. However, autologous fat tends to atrophy, which leads to volume depletion, potentially leading to remucosalization and mucocele formation. Further, it may impede imaging tests for identifying purulent complications [28, 31].

The use of hydroxyapatite cement, which is an obliteration material that has relatively high radiopacity and is easy to manipulate and distinguish, is preferred due to its capability of direct osseointegration without inducing foreign body reactions, as well as its ability to form the contour of a severely crushed anterior wall [31].

Postoperative complications may include persistent or recurrent CSF leakage, mucocele or mucopyocele formation, frontal sinusitis, frontal osteomyelitis, chronic headache, wound infection, forehead deformity, intracranial abscess, and meningitis. Mucocele formation or infection may occur if complete sinus obliteration is not achieved [30]. The reported incidence of complications has varied widely across studies, ranging from 0 to 50% [2, 3, 5, 7, 9, 10, 14, 20]. Therefore, conservative management and minimally invasive surgery have been favored in recent years to avoid complications associated with conventional surgery and preserve frontal sinus function. Choi et al. reported that the risk of persistent CSF leakage was 10% and 8% with nonsurgical and surgical treatment, respectively [7]. Ravindra et al. reported that most patients with frontal sinus injury do not require surgical intervention, and that most acute cases of post-trauma CSF rhinorrhea resolve spontaneously [12]. Patel et al. reported that contour deformity improved spontaneously and auto-reduction occurred with conservative follow-up alone [16].

Additionally, there has been a recent increase in the importance of minimizing aesthetic sequelae. With the rapid development of endoscopic instruments, endoscopic repair is being increasingly used as an alternative to traditional extracranial approaches for frontal sinus fractures and CSF leakage; further, it has gradually become the standard of care [32–36]. The endoscopic approach allows better esthetic outcomes than the existing bicoronal approach since it involves less scarring and a low risk of hair loss; furthermore, it involves a reduced risk of postoperative infection and paresthesia. However, the endoscopic approach has the disadvantages of technique sensitivity, narrow field of view, and inability for rigid fixation, especially in fractures with severe displacement [6, 37]. Alternatively, minimally invasive transcutaneous approaches, including frontal rhytid forehead incision, butterfly incision, subbrow incision, and eyebrow incision, have been designed and widely used. These approaches have the advantages of allowing direct visualization of the fracture site and rigid internal fixation [38–41].

However, severe comminuted fractures of the posterior wall of the frontal sinus are often accompanied by damage to the NFOT. This limits the use of minimally invasive surgery alone; accordingly, surgical methods using traditional extracranial approaches are still considered effective. We recommend conservative management in cases with minimal fracture site displacement and a low risk of complications. Traditional surgical management through close cooperation with relevant clinical departments is recommended for patients with severe displacement and a high risk of complications.

Among the eight patients who underwent sinus obliteration at our hospital, two patients died due to postoperative complications of intracranial infection. Cranialization had to be considered for these patients given the severe damage to the posterior wall of the frontal sinus. However, sinus obliteration was performed in these cases, which could have contributed to the death without recovery. One of the three patients who underwent cranialization presented with esthetic sequelae of the forehead and intermittent headache; however, all patients showed satisfactory recovery without any infectious complications. Despite the simplicity of the comparison and small sample size, cranialization may be preferred over sinus obliteration for patients with a high risk of intracranial infection due to severe damage to the posterior wall of the frontal sinus.

### Limitations

Our study has several limitations. First, the postoperative aesthetic outcomes were based on the patient’s subjective expression of satisfaction. Validated facial aesthetic measures, including the use of standardized photographs or radiological criteria for forehead deformities, may allow relatively accurate and objective assessment. Second, this study had a small sample size. Third, we did not include cases of endoscopic or minimal invasive transcutaneous approaches, which impeded comparative analysis with extracranial approaches. Fourth, there may be bias in our finding that cranialization is preferable over alternative treatments for severe damage to the posterior wall of the frontal sinus. Fifth, the limited follow-up period of this study may impede assessment of late complications. This is because most people may be asymptomatic and are less inclined to regularly visit the hospital. Mucocele formation or infectious complications after trauma can occur late, even after 10 years, with some cases showing occurrence as late as 35 years after trauma [7]. Therefore, continuous outpatient follow-up is necessary.

## Conclusion

Recent treatment trends for frontal sinus injuries focus on aesthetic restoration, restoration of frontal sinus function, and minimization of the incidence of intracranial complications. A multidisciplinary team approach involving neurosurgeons, oral and maxillofacial surgeons, and otolaryngologists should be considered to effectively treat these injuries. Our main protocol was to provide conservative management and follow-up observation for frontal sinus fractures without severe displacement and to perform surgery when aesthetic sequelae or infectious complications were expected due to severe displacement. Despite its limited sample size, the findings of this study can yield the following conclusions:

1. Patients with frontal sinus fractures without severe displacement achieved good treatment outcomes with only follow-up observation and conservative management, without the incidence of any special complications.
2. Surgical treatment through the opening of the frontal sinus is preferred for patients with a high risk of intracranial infection due to severe damage to the posterior wall of the frontal sinus.

## Data Availability

Not applicable.

## Abbreviations

CSF: Cerebrospinal fluid
NFOT: Nasofrontal outflow tract
CT: Computed tomography
MRI: Magnetic resonance imaging
SDH: Subdural hematoma
SAH: Subarachnoid hemorrhage
ICH: Intracerebral hemorrhage
EDH: Epidural hematoma
